# Tomato consumption and prostate cancer risk: a systematic review and meta-analysis

**DOI:** 10.1038/srep37091

**Published:** 2016-11-14

**Authors:** Xin Xu, Jiangfeng Li, Xiao Wang, Song Wang, Shuai Meng, Yi Zhu, Zhen Liang, Xiangyi Zheng, Liping Xie

**Affiliations:** 1 Department of Urology, First Affiliated Hospital, School of Medicine, Zhejiang University, Hangzhou, 310003, China

## Abstract

Previous studies have reported controversial results on the association between tomato consumption and prostate cancer risk. Hence, we performed a meta-analysis to comprehensively evaluate this relationship. A total of 24 published studies with 15,099 cases were included. Relative risks (RR) and 95% confidence intervals (CI) were pooled with a random-effects model. Tomato intake was associated with a reduced risk of prostate cancer (RR 0.86, 95% CI 0.75–0.98, *P* = 0.019; *P* < 0.001 for heterogeneity, *I*^*2*^ = 72.7%). When stratified by study design, the RRs for case-control and cohort studies were 0.76 (95% CI 0.61–0.94, *P* = 0.010) and 0.96 (95% CI 0.84–1.10, *P* = 0.579), respectively. In the subgroup analysis by geographical region, significant protective effects were observed in Asian (RR 0.43, 95% CI 0.22–0.85, *P* = 0.015) and Oceania populations (RR 0.81, 95% CI 0.67–0.99, *P* = 0.035), but not in other geographical populations. Begg’s test indicated a significant publication bias (*P* = 0.015). Overall, tomato intake may have a weak protective effect against prostate cancer. Because of the huge heterogeneity and null results in cohort studies, further prospective studies are needed to explore the potential relationship between tomato consumption and prostate cancer risk.

Emerging evidence from epidemiological, as well as cell culture and animal, studies indicates that lycopene and the consumption of lycopene-containing foods may be protective against cancer and cardiovascular disease risk^1^, notably stroke^2^, hypertension^3^, and prostate cancer^4^,^5^.

Processed tomato products are the primary dietary lycopene source^6^. The association between tomato food and prostate cancer has been investigated by numerous epidemiological studies, with inconsistent results. Some reported that individuals with higher intake of tomato foods had a lower risk of prostate cancer compared with consumers of lower tomato intake^7^,^8^,^9^,^10^,^11^,^12^,^13^,^14^,^15^, while others found null results^16^,^17^,^18^,^19^,^20^. Darlington *et al*.^21^ even reported a positive association between consumption of tomato and incidence of prostate cancer.

A previous meta-analysis published in 2004 reported that tomato consumption might play a protective role in the prevention of prostate cancer based on three cohort and seven case-control studies^22^. However, a latest meta-analysis of seven cohort studies from the World Cancer Research Fund (2014) failed to confirm this association^23^. The overall purpose of the present study was to evaluate the strength of this controversial association, by performing a systematic review and meta-analysis of all eligible cohort and case-control studies published on the subject in peer-reviewed literature up to now. In addition, we performed a stratified analysis by geographical region to explore the potential regional differences.

## Results

### Literature search and study characteristics

**Figure 1:**
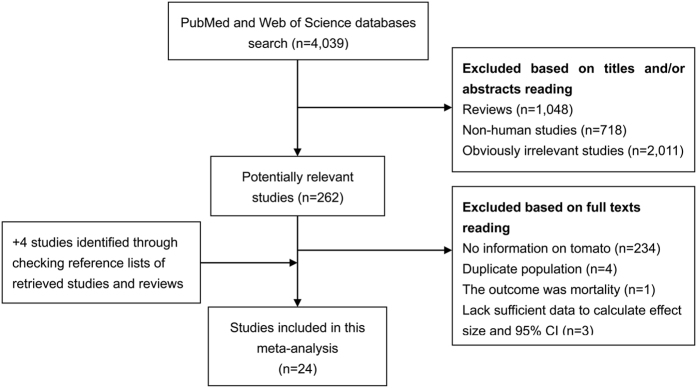
Process of literature search and study selection.

Figure 1 presents the detailed process of literature review. A total of 24 eligible studies^7^,^8^,^9^,^10^,^11^,^12^,^13^,^14^,^15^,^16^,^17^,^18^,^19^,^20^,^21^,^24^,^25^,^26^,^27^,^28^,^29^,^30^,^31^,^32^ were eventually included in this meta-analysis aimed to comprehensively evaluate the relationship between tomato intake and prostate cancer risk. There were 7 cohort and 17 case-control studies, which were performed in the following geographical regions: Europe (n = 4), North America (n = 10), Asia (n = 7), and Oceania (n = 3). Up to 15,099 cases were analyzed in these studies published between 1989 and 2016. Data on exposure (tomato intake) was mainly collected by interview or questionnaire and outcome (prostate cancer) was confirmed histologically in the majority of the included studies. The study quality was evaluated by the Newcastle-Ottawa Scale (NOS). Scores ranged from 5 to 8, with a mean of 6.08. Table 1 summaries the main characteristics of all included studies analyzed in this meta-analysis.

### Pooled analysis and heterogeneity assessment

**Figure 2:**
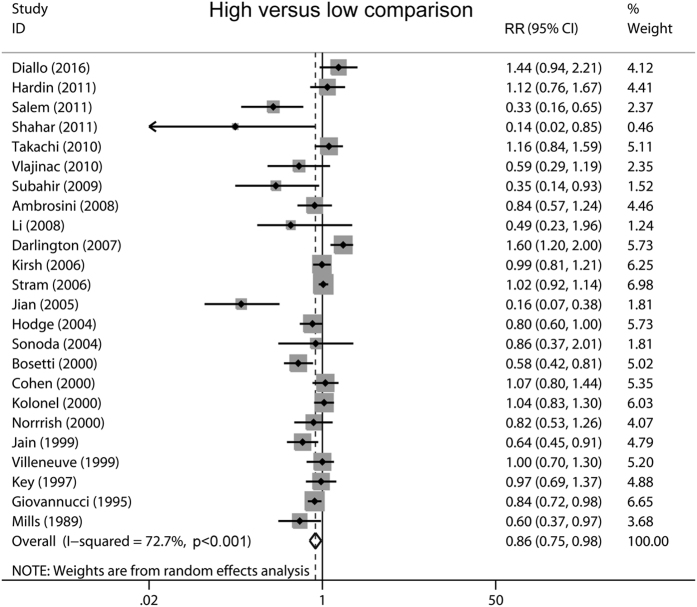
Overall analysis of the association between tomato consumption and prostate cancer risk.

Multivariable adjusted relative risks (RRs) with their confidence intervals (CIs) for each individual study and for the combination of all included studies are shown in Fig. 2. In a random-effect pooled analysis of these studies, high-tomato intake (comparing the highest with the lowest category) was associated with a reduced prostate cancer risk (RR 0.86, 95% CI 0.75–0.98, *P* = 0.019). Statistically significant heterogeneity was observed among included studies (*P* < 0.001 for heterogeneity, *I*^*2*^ = 72.7%).

### Subgroup analysis

**Figure 3:**
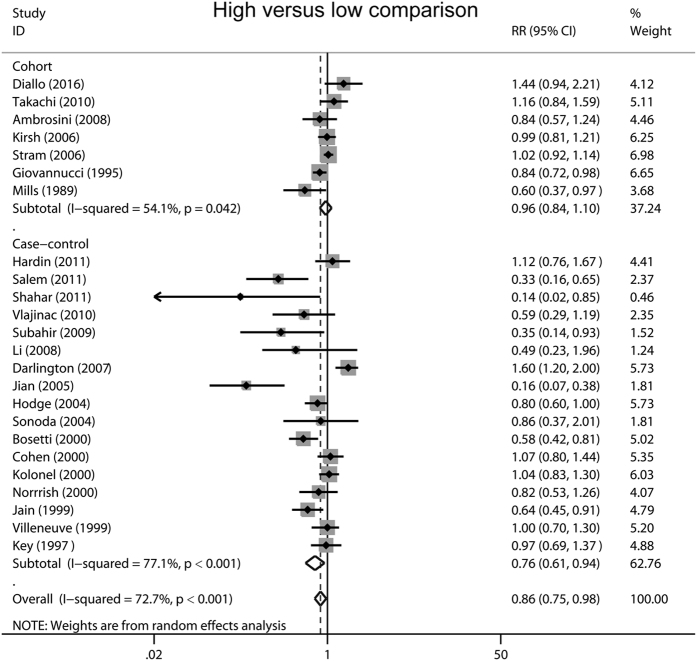
Forest plots showing risk estimates from case-control and cohort studies estimating the association between tomato consumption and prostate cancer risk.

The effects of tomato intake on prostate cancer risk in subgroup meta-analyses are shown in Table 2. We firstly performed stratified analyses by geographical region, significant protective effects of tomato intake against prostate cancer were observed in Asian (RR 0.43, 95% CI 0.22–0.85, *P* = 0.015) and Oceania populations (RR 0.81, 95% CI 0.67–0.99, *P* = 0.035), but the effects were not significant in other geographical populations. When stratified by study design, the analysis of case-control studies yielded a RR of 0.76 (95% CI 0.61–0.94, *P* = 0.010), whereas the analysis based on cohort studies yielded a RR of 0.96 (95% CI 0.84–1.10, *P* = 0.579) (Fig. 3). In the subgroup analysis by study quality, more pronounced association was detected in studies with low quality (RR 0.77, 95% CI 0.61–0.98, *P* = 0.030) compared with high-quality studies (RR 0.92, 95% CI 0.79–1.06, *P* = 0.234). Finally, in the stratified analyses by sample size, statistically significant association was observed in those small studies (RR 0.69, 95% CI 0.54–0.89, *P* = 0.005) rather than in large studies (RR 0.98, 95% CI 0.86–1.12, *P* = 0.763).

### Sensitivity analysis and publication bias

**Figure 4:**
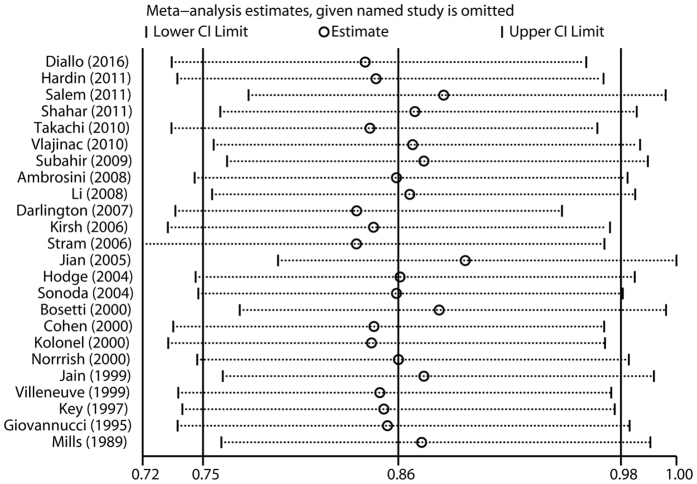
Sensitivity analysis was performed whereby each study was excluded in turn and the pooled estimate recalculated to determine the influence of each study.

The influence of each study on the pooled RR was evaluated by repeating the overall analysis after omitting each study in turn. The results indicated that no single study dominated the combined RR. The 24 study-specific RRs ranged from a low of 0.83 (95% CI 0.72–0.97) to a high of 0.89 (95% CI 0.79–1.00) via omission of the study by Stram *et al*.^20^ and the study by Jian *et al*.^11^, respectively (Fig. 4). Finally, significant publication bias was observed in Begg’s test (*P* = 0.015), but not in Egger’s test (*P* = 0.122).

## Discussion

This systematic review and meta-analysis aimed to evaluate the relationship between tomato intake and prostate cancer risk based on 7 cohort studies and 17 case-control studies, with a total of 15,099 cases. The results of this quantitative meta-analysis provided limited evidence for a protective effect of high tomato food consumption for prostate cancer incidence. Although the overall analysis suggested a moderate reduction in risk, the results from the cohort, high-quality, and large studies were null.

The findings of this meta-analysis are basically consistent with a previous meta-analysis published in 2004^22^, which included three cohort and seven case-control studies. Its results also indicated that tomato consumption might play a protective role in the prevention of prostate cancer. But the effect was modest and restricted to high amounts of tomato intake^22^. Since then, emerging studies on this topic have been published, while the results were still conflict. In 2014, a meta-analysis of seven cohort studies from the World Cancer Research Fund reported no significant association between tomato intake and prostate cancer risk. The combined RR per 1 serving/day was 0.93 (95% CI 0.79–1.09; *I*^*2*^ = 52.0%)^23^. Similarly, when stratified by study design in this study, the analysis based on cohort studies yielded a RR of 0.96 (95% CI 0.84–1.10, *I*^*2*^ = 54.1). Therefore, a protective effect of tomato intake on the risk of prostate cancer is mainly observed in case-control studies. Compared with these previous meta-analyses, the present updated meta-analysis also performed a stratified analysis by geographical region, which provided a more comprehensive assessment of the association between tomato consumption and prostate cancer risk.

Several potential mechanisms could explain the potential cancer-protective effects of tomato food. Tomato food has high levels of lycopene, which has been shown to inhibit prostate cancer progression in several studies. Yang *et al*.^33^ reported that lycopene could suppress the proliferation of androgen-dependent human prostate tumor cells (LNCaP) through activation of PPARγ-LXRα-ABCA1 pathway. Elgass *et al*.^34^ found that lycopene could also inhibit the cell adhesion and migration properties in androgen-independent prostate cancer cells (PC3 and DU145). *In vivo* studies, dietary tomato and lycopene could have an influence on androgen signaling- and carcinogenesis-related gene expression during early transgenic adenocarcinoma of the mouse prostate (TRAMP) mice prostate carcinogenesis^35^. In epidemiological studies, lycopene consumption (both dietary intake and its blood levels) has been linked to a reduced risk of prostate cancer^4^.

This study had several important strengths. First, as individual studies may have limited statistical power, our meta-analysis of 24 published studies with 15,099 prostate cancer cases might provide more reliable results with greater precision and power. Second, we extracted data from the most fully adjusted model in each study, which reduce the potential influence of confounding factors. Third, various subgroup analyses, influence analysis, and publication bias analysis were performed to evaluate the robustness of the pooled risk estimate.

However, several limitations should be considered in interpreting the results of this meta-analysis. First, there was substantial heterogeneity across studies (*P* < 0.001 for heterogeneity, *I*^*2*^ = 72.7%), which was likely due to the variation in population information, exposure definitions, exposure ranges, exposure and outcome assessment methods between studies. Second, Begg’s test suggested the existence of publication bias. Although we adopted a loose search strategy, some inevitable publication bias might exist as small studies with negative results were less likely to be published and gray literature (such as non-English articles and conference abstract) was difficult to find. Third, the cutoff points for the lowest and highest categories of the tomato intake were various in included studies, which might also has an influence on the combined risk estimate. Finally, the association between lifestyle factors and prostate cancer risk may vary by tumor characteristics (e.g., stage and grade). However, most of the included studies didn’t provide risk estimates for localized/low grade and advanced/high grade cancers separately. Therefore, we were not able to examine if there were differences by stage and grade in the association between tomato intake and prostate cancer risk.

## Conclusion

In summary, this meta-analysis indicates that tomato intake may be associated with a reduced risk of prostate cancer. The significant protective effects were observed in Asian and Oceania populations, but not in other geographical populations. As there were no significant results in cohort and high-quality studies, no firm conclusions can be drawn at the present time. Further large-scale prospective cohorts, as well as mechanistic studies, are needed to clarify the relationship between tomato food intake and prostate cancer risk.

## Materials and Methods

### Literature review

A comprehensively literature search of published articles was performed in June 2016 based on PubMed and Web of Science databases. We found that few studies were eligible when only using “tomato” and “prostate cancer” as search terms. Therefore, we adopted the following loose search algorithm: (“diet” or “nutrition” or “vegetable” or “vegetables” or “tomato” or “tomatoes” or “lycopene”) and (“prostatic neoplasms” or “prostatic cancer” or “prostate neoplasms” or “prostate cancer”). Furthermore, the cited references of retrieved articles and reviews were also checked to identify any additional relevant studies. There was no language, publication date, or publication status restrictions. This systematic review and meta-analysis was designed, performed, and reported in accordance with the standards of quality for reporting meta-analyses, except for not publishing the review protocol in advance^36^.

### Study selection criteria

A study was included if it met the following criteria: (*i*) the exposure of interest was consumption of tomato food; (*ii*) the outcome of interest was incidence of prostate cancer; (*iii*) study design was cohort, nested case-control or case-control; and (*iv*) the effect sizes with their corresponding 95% CIs were reported. If multiple articles reported data based on the same population, the publication with the most up-to-date or comprehensive information was included in the meta-analysis.

### Study quality assessment

A 9-star system on the basis of the NOS (http://www.ohri.ca/programs/clinical_epidemiology/oxford.asp) was used to assess the quality of each included study by two independent reviewers (XX and JFL). NOS judges a study according to the following three broad perspectives: selection (four items), comparability (one item), and exposure/outcome (three items). Each item is awarded one point, except for comparability (two points). Hence, the full score is 9 stars. A study with ≥7 awarded stars is classified as high quality.

### Data extraction

Information was collected and recorded independently by two investigators (XX and JFL). Any discrepancies were resolved through iteration and consensus. The following data were obtained from each study: first author’s surname, country, publication year, study design, age, number of cases, instrument of exposure measurement, method of outcome assessment, results of studies (adjusted risk estimates with their corresponding 95% CIs), and matched or adjusted confounding factors in the design or statistical analysis.

### Statistical methods

Considering that prostate cancer is a rare disease, the odds ratio (OR) was assumed approximately the same as RR, and the RR was designated as the study outcome. Multiple adjusted RRs with their 95% CIs were used to measure the strength of the relationship between tomato intake and prostate cancer risk. Some studies reported risk estimates for raw tomato and cooked tomato separately and did not report the effect of total tomato intake. In this situation, the study-specific RR in overall analysis was recalculated by pooling the risk estimates with the inverse-variance method^37^. A DerSimonian and Laird random-effects model^38^, which incorporates both within- and between-study variability, was applied to calculate the combined RR and its 95% CI. Subgroup analyses were carried out by geographical region, study design, study quality, and sample size.

Statistical heterogeneity among included studies was estimated using Cochran’s *Q* test and the *I*^*2*^ score^39^. The level of significancefor Cochran’s *Q* was test set at 0.1 (10%). The *I*^*2*^ score was adopted to evaluate the degree of heterogeneity (*I*^*2*^ < 25%: no heterogeneity; *I*^*2*^ = 25–50%: moderate heterogeneity; *I*^*2*^ > 50%: large or extreme heterogeneity).

A sensitivity analysis was conducted by omitting each study in turn and recalculating the pooled RR to test the impact of each study on the overall risk estimate. Potential publication bias was assessed through Begg’s test (rank correlation method)^40^ and Egger’s test (linear regression method)^41^. All statistical analyses were conducted with STATA 11.0 (StataCorp, College Station, TX), using two-sided *P* values (set at 0.05).

## Additional Information

**How to cite this article**: Xu, X. *et al*. Tomato consumption and prostate cancer risk: a systematic review and meta-analysis. *Sci. Rep.* **6**, 37091; doi: 10.1038/srep37091 (2016).

**Publisher’s note**: Springer Nature remains neutral with regard to jurisdictional claims in published maps and institutional affiliations.

## Figures and Tables

**Table 1:** Characteristics of the studies included in this meta-analysis.

Author	Year	Region	Design	No. of cases	Age (yr)	Exposure assessment	Outcome assessment	Matched or adjusted factors	NOS score
Diallo *et al*.	2016	France	Cohort	139	63	Interview	Biopsy	Age, energy intake, intervention group of the initial SU.VI.MAX trial, number of 24-h dietary records, smoking, education, physical activity, height, BMI, alcohol, family history of prostate cancer, baseline plasma PSA, Ca intake, dairy product intake and plasma α-tocopherol and Se concentrations	8
Hardin *et al*.	2011	USA	Case-control	470	65.8 (SD 8.3)	Questionnaire	Histologically confirmed	Age, race, institution, energy intake, and history of first-degree relative with prostate cancer	6
Salem *et al*.	2011	Iran	Case-control	194	71.1 (SD 7.84)	Interview	Histologically confirmed	Age, total dietary calories, BMI, occupation, education, smoking, alcohol, and family history of prostate cancer.	7
Shahar *et al*.	2011	Malaysia	Case-control	35	67.6 (SD 4.7)	Interview	Biopsy	Age, ethnic, family history of cancer, and energy intake	5
Takachi *et al*.	2010	Japan	Cohort	339	40–69	Questionnaire	Cancer registry	Age, public health center area, BMI, smoking, alcohol, dairy food, soy products, green tea, vitamin supplement use, marital status, screening examination	6
Vlajinac *et al*.	2010	Serbia	Case-control	101	NA	Questionnaire	Histologically confirmed	Age, hospital admission, place of residence, and energy	5
Subahir *et al*.	2009	Malaysia	Case-control	112	71.7 (50–86)	Questionnaire	Histologically confirmed	Age and ethnicity	5
Ambrosini *et al*.	2008	Australia	Cohort	97	62.6	Questionnaire	Cancer registry	Age, total fruit and vegetable intake, randomly assigned retinol or β-carotene supplement, and source of crocidolite exposure	6
Li *et al*.	2008	China	Case-control	28	71.4 (SD 6.0)	Interview	Biopsy	Age, place of employment, education, BMI, smoking, alcohol, and food frequency	5
Darlington *et al*.	2007	Canada	Case-control	752	50–84	Questionnaire	Cancer registry	Age, family history of prostate cancer, BMI, education, type of occupation, and total energy	6
Kirsh *et al*.	2006	USA	Cohort	1338	63.3	Questionnaire	Medical/pathologic records	Age, total energy, race, study center, family history of prostate cancer, BMI, smoking, physical activity, supplemental vitamin E, total fat, red meat, history of diabetes, aspirin use, and previous number of screening exams	7
Stram *et al*.	2006	USA	Cohort	3922	45–75	Questionnaire	SEER registry	Age, BMI, education, and family history of prostate cancer	7
Jian *et al*.	2005	China	Case-control	130	72.7 (SD 7.1)	Questionnaire	Histologically confirmed	Age, locality, education, family income, marital status, number of children, family history of prostate cancer, BMI, tea drinking, caloric intake, and fat intake	5
Hodge *et al*.	2004	Australia	Case-control	858	<70	Interview	Histologically confirmed	Age, state, year, country of birth, socioeconomic group, total energy intake, and family history of prostate cancer	6
Sonoda *et al*.	2004	Japan	Case-control	140	59–73	Questionnaire	Histologically confirmed	Age, smoking, and energy intake.	5
Bosetti *et al*.	2000	Greece	Case-control	320	NA	Questionnaire	Histologically confirmed	Age, height, BMI, years of schooling, total energy intake, milk and dairy products, butter, and seed oils intake	5
Cohen *et al*.	2000	USA	Case-control	628	40–64	Questionnaire	Histologically confirmed	Age, fat, energy, race, family history of prostate cancer, BMI, PSA tests, education, and total vegetables	7
Kolonel *et al*.	2000	USA	Case-control	1619	≤84	Interview	Histologically confirmed	Age, education, ethnicity, geographic area, and calories	6
Norrrish *et al*.	2000	New Zealand	Case-control	317	40–80	Questionnaire	Histologically confirmed	Age, height, total NSAIDs, and socioeconomic status	7
Jain *et al*.	1999	Canada	Case-control	617	69.8	Interview	Cancer registry	Age, total energy, vasectomy, ever-smoked, marital status, study area, BMI, education, multivitamin supplements, area of study, and log-converted amounts for grains, fruit, vegetables, total plants, total carotenoids, folic acid, dietary fiber, conjugated linoleic acid, vitamin E, vitamin C, retinol, total fat, and linoleic acid	7
Villeneuve *et al*.	1999	Canada	Case-control	1623	50–74	Questionnaire	Histologically confirmed	Age, province of residence, race, years since quitting smoking, cigarette pack-years, BMI, rice and pasta, coffee, grains and cereals, alcohol, fruit and fruit juices, tofu, meat, income, and family history of cancer	7
Key *et al*.	1997	UK	Case-control	328	68.1	Questionnaire	Histologically records	Age and social class	6
Giovannucci *et al*.	1995	USA	Cohort	812	40–75	Questionnaire	Medical records	Age and energy	7
Mills *et al*.	1989	USA	Cohort	180	74	Questionnaire	Histologically confirmed	Age, education, current use of meat, poultry, or fish, current fish only, beans, legumes or peas, citrus fruit, dry fruit, and index of fruit, nuts	5

No., number; NOS, Newcastle-Ottawa Scale; yr, year; SD, standard deviation; BMI, body mass index; PSA, prostate-specific antigen; NSAIDs, non-steroidal anti-inflammatory drugs; NA, not available.

**Table 2:** Subgroup analyses of the association between tomato intake and prostate cancer risk.

Subgroup	Included studies	No. of cases	Pooled RR (95% CI)	*P*	Heterogeneity		
					Q	*I*^*2*^ (%)	*P*
**Total**	24	15,099	0.86 (0.75-0.98)	0.019	84.29	72.7	< 0.001
**Study design**							
Cohort	7	6,827	0.96 (0.84-1.10)	0.579	13.06	54.1	0.042
Case-control	17	8,272	0.76 (0.61-0.94)	0.010	69.83	77.1	< 0.001
**Geographical region**							
North America	10	11,961	0.98 (0.86-1.13)	0.811	29.11	69.1	0.001
Europe	4	888	0.85 (0.55-1.31)	0.455	12.63	76.3	0.006
Asia	7	978	0.43 (0.22-0.85)	0.015	31.29	80.8	< 0.001
Oceania	3	1,272	0.81 (0.67-0.99)	0.035	0.04	0.0	0.978
**Study quality**							
High (NOS ≥ 7)	9	9,590	0.92 (0.79-1.06)	0.234	22.69	64.7	0.004
Low (NOS < 7)	15	5,509	0.77 (0.61-0.98)	0.030	61.40	77.2	< 0.001
**No. of cases**							
≥500	9	12,169	0.98 (0.86-1.12)	0.763	27.21	70.6	0.001
<500	15	2,930	0.69 (0.54-0.89)	0.005	49.44	71.7	< 0.001

No., number; RR, relative risk; CI, confidence interval; NOS, Newcastle-Ottawa Scale.
